# Testing robustness of relative complexity measure method constructing robust phylogenetic trees for *Galanthus* L. Using the relative complexity measure

**DOI:** 10.1186/1471-2105-14-20

**Published:** 2013-01-17

**Authors:** Yasin Bakış, Hasan H Otu, Nivart Taşçı, Cem Meydan, Neş’e Bilgin, Sırrı Yüzbaşıoğlu, O Uğur Sezerman

**Affiliations:** 1 Department of Biology, Abant İzzet Baysal University, Bolu, 14280, Turkey; 2 Department of Medicine, BIDMC Genomics Center, Harvard Medical School, Boston, MA, 02115, USA; 3 İstanbul Bilgi University, Department of Genetics and Bioengineering, Eyüp, İstanbul, 34060, Turkey; 4 Department of Molecular Biology and Genetics, Boğaziçi University, Bebek, İstanbul, 34342, Turkey; 5 Biological Sciences and Bioengineering, Sabancı University, Tuzla, İstanbul, 34956, Turkey; 6 Department of Botany, İstanbul University, Süleymaniye, İstanbul, 34460, Turkey

**Keywords:** Resampling, Alignment free, Phylogenetics, Relative Complexity Measure, Galanthus

## Abstract

**Background:**

Most phylogeny analysis methods based on molecular sequences use multiple alignment where the quality of the alignment, which is dependent on the alignment parameters, determines the accuracy of the resulting trees. Different parameter combinations chosen for the multiple alignment may result in different phylogenies. A new non-alignment based approach, Relative Complexity Measure (RCM), has been introduced to tackle this problem and proven to work in fungi and mitochondrial DNA.

**Result:**

In this work, we present an application of the RCM method to reconstruct robust phylogenetic trees using sequence data for genus *Galanthus* obtained from different regions in Turkey. Phylogenies have been analyzed using nuclear and chloroplast DNA sequences. Results showed that, the tree obtained from nuclear ribosomal RNA gene sequences was more robust, while the tree obtained from the chloroplast DNA showed a higher degree of variation.

**Conclusions:**

Phylogenies generated by Relative Complexity Measure were found to be robust and results of RCM were more reliable than the compared techniques. Particularly, to overcome MSA-based problems, RCM seems to be a reasonable way and a good alternative to MSA-based phylogenetic analysis. We believe our method will become a mainstream phylogeny construction method especially for the highly variable sequence families where the accuracy of the MSA heavily depends on the alignment parameters.

## Background

Plenty of phylogenetic analysis techniques exist showing relations among organisms based on molecular sequences. Most commonly utilized methods are distance based methods and evolutionary methods [1,2]. Distance based methods, such as Neighbor-Joining [3] and UPGMA [4], explicitly rely on a measure of genetic distance between OTUs based on their sequence differences. Distance measures are derived from pairwise comparisons of the sequences. Whereas the distance based methods represent sequence divergence by a single number, the evolutionary methods attempt to infer the phylogeny by fitting individual characters (nucleotides or amino acids) to the tree. Most popular approaches for evolutionary methods are maximum likelihood [5], maximum parsimony [6,7], and Bayesian inference [8]. These methods compare various evolutionary trees that could describe the relationships among given taxa. The tree that implies the fewest or most likely evolutionary changes in the characters is taken to be the best estimate of the true phylogenetic tree. A new method in phylogeny reconstruction, namely, simultaneous sequence alignment has recently been introduced by [9]. The method is particular in that it can perform true alignment and phylogenetic inference. Mutations can then be included in the overall tree score.

In most distance based and evolutionary methods, one must perform a multiple sequence alignment (MSA) first to infer the relationships between the given set of molecular sequences. MSA is still a notoriously difficult computational problem and different tools often produce different alignments [10,11]. This leaves users with the problem of choosing the most appropriate alignment method for their use. Distance based or character based phylogeny construction methods that rely on an MSA can lead to misleading trees due to errors in the alignment [12]. First of all, when more divergent sequences are to be incorporated, generating reliable multiple alignments becomes increasingly difficult [13]. The second problem is the difficulty in the choice of suitable scoring matrices and gap penalties for different sequence sets [6,7]. Another major problem arises when progressive MSA schemes – which are increasingly popular – are used such as CLUSTALW [14], T-Coffee [15] and MAFFT [16]. In such schemes, the reliance of the final MSA is mostly based on a good alignment of the first two sequences which are generally the most closely related sequences [14]. If the error in the alignment of the more closely related sequences cannot be corrected, the error will be increasingly serious within the rest of the alignment procedures. Another disadvantage is that the number of sequences is limited. The computing time complexity is proportional to the exponential of the number of the sequences to be analyzed [9,14,17].

To overcome these problems, alignment-free phylogeny construction seems to be a reasonable way. Application of the non-alignment based methods has gained popularity especially in the analysis of the genome-wide sequences where MSA can hardly be utilized. Relative Complexity Measure (RCM) method [18,19] has been proposed as a sequence distance measure with applications to phylogeny construction based on Lempel-Ziv (LZ) complexity [20]. It is an alignment free method, since it does not require a preceding multiple alignment procedure and can be grouped within the distance based methods. Although the phylogenies generated by RCM were found highly reasonable [18,19,21] the statistical significance of the trees have not been tested in detail. Resampling techniques utilizing bootstrap have been used in phylogenetic analyses to assess statistical significance of the tree topology: complete and partial bootstrap [22-24], block bootstrap [25], jackknife; delete-half [22] and delete fraction [26], permuting species or characters [27]. However, most of these methods are based on data with equal-size samples or require a preceding alignment procedure. Since RCM does not rely on alignment, we propose a perturbance technique mimicking the evolutionary process in order to test the robustness of the trees obtained by RCM. We demonstrate the accuracy of Relative Complexity Measure method using different sets of benchmark sequence families generated by ROSE [28], a probabilistic model of the evolution of DNA sequences. In this study, robustness of the RCM approach was assessed on *Galanthus* by comparing RCM results to other phylogeny construction methods. Source of the molecular sequences were from genetic material of two different cell organs telling different evolutionary stories.

### Galanthus

*Galanthus* L., widely known as snowdrops, belongs to the family Amaryllidaceae. It is a genus of bulbous monocotyledons, consisting of 19 species confined to Europe, Asia Minor, and the Near East. Taxonomy of *Galanthus* is clarified by [29] (Table 1). Turkey is one of the centers of species diversity and is home to 16 recognized *Galanthus* species and some are endemic to Anatolia. However, the taxonomic status and the identity of some of the species in Turkey are still unclear. 

**Table 1:** Comparison of average symmetric distances between true topology and constructed phylogeny for simulated data with standard deviations in parenthesis

	**K2P**		**F84**	
DNADist	0.211	(0.65)	0.219	(0.65)
DNAML	0.179	(0.62)	0.168	(0.60)
POY	0.149	(0.41)	0.175	(0.45)
RCM	0.114	(0.47)	0.149	(0.54)
PhyML	0.163	(0.59)	0.174	(0.59)

Due to the pressure of humankind, the survival of many *Galanthus* species is threatened in nature and in most countries it is now forbidden to remove *Galanthus* from the wild, as they are usually protected by local laws [30]. Inspections of the *Galanthus* bulb trade require the difficult task of determining the taxonomy of the collected bulbs. Studies on developing such methods would also provide useful data to be used in the identification of newly collected *Galanthus* species. The molecular karyotypic properties of the whole genera are found to be similar in [31,32]. The nuclear DNA content of different diploid *Galanthus* species, analyzed through flow cytometry, show a low intraspecific variation [33]. A group of scientists from Leiden University proposed the measurement of nuclear DNA content as a rapid and cost-effective tool for identifying wild origin of *Galanthus* species [33].

## Methods

### Simulated data

To test the accuracy of Relative Complexity Measure method we have used benchmark sequence families generated by Random Model of Sequence Evolution (ROSE version 1.3) [28]. The test was performed on the balanced phylogeny – phylogeny ‘a’ in [34,35] – with 16 OTUs. For two DNA models, F84 and K2P, 966 and sets of sequence families were generated respectively. The DNA sequences have an average length of 500 letters as indicated in [34] and highest value of relatedness (1000) was given to show the symmetric distances between the topologies. Default values were used for the rest of the parameters. By this way, 2058 sequence families were collected.

The correct multiple sequence alignment is created by ROSE simultaneously, since the true history of evolutionary process is logged. Phylogenies of collected sets of sequences were constructed by POY [36,37], DNADist, DNAML [38], PhyML [39], and RCM [18] methods and compared. The DNA models that were used to generate sequence families were also given as a parameter within DNADist. The phylogenies for DNADist and RCM methods were calculated by NEIGHBOR routine of Phylogeny Inference Package (PHYLIP version 3.67) [40] with default parameters, and the topology (symmetric) distance measure [41] of TREEDIST routine was used to calculate the distances between constructed phylogenies and the true phylogeny.

## Real data

### Collection of specimens

All plant samples were collected at the time they are flowering and were identified according to their morphological features. At least one bulb per location was obtained and leaves of the plants were used as material for molecular analysis [42].

### Molecular analysis

Nuclear and chloroplastic DNA sequences were obtained from the same individual. Fresh leaves were cut into small pieces and quickly grinded to a fine powder in liquid nitrogen and preserved at −80°C until further use. DNA from frozen powdered tissue was extracted using Qiagen Plant DNA Extraction Minikit, following manufacturer’s instructions. DNA isolates were sequenced and submitted to GenBank with accession numbers GU329529-GU329704.

### PCR amplification of nuclear ribosomal RNA ITS region

**Figure 1:**
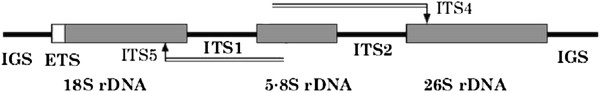
**Nuclear ribosomal RNA ITS region amplified by PCR for sequencing.** Arrows indicate the positions of the forward ITS5 primer and the reverse ITS4 primer used for this amplification [42].

The amplification of the intragenic spacer region (ITS1-5.8S-ITS2 rDNA) between the ribosomal RNA genes was performed using the universal primers designed by [43]. The amplified DNA region is shown in Figure 1. 

The PCR reaction mix in 100 μL contained 1X PCR buffer, 1.5 mM MgCl_2_, 0.2 mM of each dNTP, 1 μM of each primer, 200 ng of DNA and 1.25U of GoTaq Flexi DNA Polymerase (Promega). PCR cycles were as follows: 94°C for 150 sec for initial denaturing, then 30 cycles of 95°C for 30 sec, 52°C for 90 sec, 72°C for 180 sec, followed by a final extension at 72°C for 7 minutes.

### PCR amplification of the chloroplast markers

**Figure 2:**
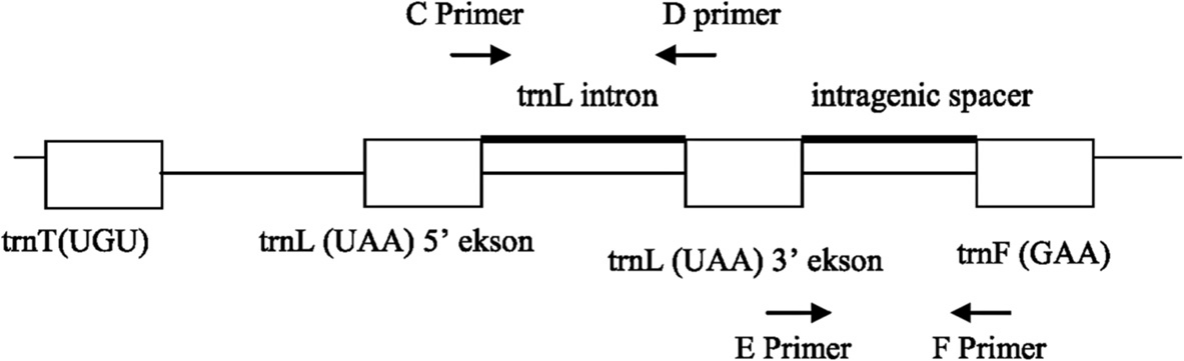
**Chloroplast DNA region amplified by PCR for DNA sequencing **[42].

The two non-coding regions of the chloroplast DNA, *trnL*(UAA) intron and the spacer between the *trnL*(UAA) 3’ and *trnF*(GAA) 5’ genes were amplified using the universal primers designed from conserved chloroplast tRNA gene sequences [44]. The positions of primers are shown in Figure 2. Primers C and D were used as forward and reverse primers, respectively for the amplification of the *trnL*(UAA) intron; primers E and F were used as forward and reverse primers, respectively for the amplification of the intergenic spacer between the *trnL*(UAA) 3’ exon and *trnF*(GAA) gene. 

The PCR reaction mix in 100 μL contained 1X PCR buffer, 1.5 mM MgCl_2_, 0.2 mM of each dNTP, 1 μM of each primer, 200 ng of DNA and 1.25U of Go Taq Flexi DNA Polymerase (Promega). PCR cycles were as follows: 94°C for 150 sec for initial denaturation, followed by 30 cycles of 95°C for 30 sec, 52°C for 90 sec, 72°C for 180 sec, followed by a final extension at 72°C for 7 min. PCR products were purified by using Wizard SV PCR Clean-Up system (Promega) before DNA sequencing.

### DNA sequencing

DNA Sequencing was carried out using DYEnamic ET Terminator Cycle Sequencing Kit (Amersham Biosciences, USA). For each sequencing reaction 3 μL of purified PCR product was added to the reaction mix containing 5 pmol of primer, 8 μL of sequencing reagent premix in a total volume of 20 μL. Each PCR product was sequenced twice using the forward and the reverse primers separately. The cycle sequencing was done on ABI 9700 Thermocycler (Applied Biosystems) with 25 cycles of 95°C for 20 s, 50°C for 15 s and 60°C for 60 s. After cycle sequencing, the unbound dyes were removed by DyeEx 2.0 Dye Removal Kit (Qiagen). The purified products were analyzed on the ABI 3100 Genetic Analyzer (Applied Biosystems).

DNA sequencing data for nuclear ribosomal RNA intragenic spacer regions ITS1, ITS2 and 5.8S rRNA) and chloroplast intergenic spacer between *trn*L(UAA) 3’ and *trn*F(GAA) 5’ genes including *trn*L(UAA) intron sequences were used from [42]. ITS region has preferably been used in the study because of its relatively fast evolutionary rate and its easy amplification using universal primers [45]. Chloroplast sequences were used to compare nuclear DNA-based phylogenies with maternally inherited chloroplast sequence-based phylogenies.

ITS sequences were varying in size ranging from 506 to 517 bp and chloroplast sequences vary in size from 804 to 817 bp (Table 1). ITS sequences were sampled from 16 species of genus *Galanthus* which are (Series *Latifolii* Subseries *Glaucaefolii*) *G. peshmenii, G. cilicicus, G. gracilis, G. elwesii var. elwesii, G. elwesii var. monostictus, G. alpinus var. alpinus,* (Series *Latifolii* Subseries *Viridifolii*) *G. rizehensis, G. woronowii, G. fosteri, G. krasnovii,* (Series *Galanthus*) *G. nivalis, G. plicatus ssp. plicatus, G. plicatus subsp. byzantinus,* (Naturally occurring hybrids) *G. xvalentinei nothosubsp subplicatus,* (Species of uncertain affinity) *G. trojanus* claimed with synopsis in [29] according to morphological observations. Chloroplast sequences were from the same 15 species of the same genus except *G. trojanus*. *Sternbergia lutea* was chosen as outgroup species.

## Phylogenetic analysis

### Perturbance technique

An algorithm has been developed for nucleotide mutation that would provide degeneration to create perturbance from the original dataset. The algorithm generated such samples by mutating randomly chosen k% of the bases of the original sequence via equal instances of insertion, deletion and substitution mimicking the evolutionary process. Bases that were removed from the sequence by deletion process were collected into a pool, afterward exchanged with bases from sequence by substitution, finally all bases within the pool inserted into the sequence separately. We introduced mutations accounting for 1%, 2%, 5%, 10%, 15%, 20% and 25% of the original sequence. This procedure has been repeated for 1000 times for each sequence to create any of the k% degenerations in the study.

A thousand datasets were created by perturbation as recommended for most resampling studies by [46]. For nuclear ITS and chloroplast data, perturbed data sets have been created at different mutation rates to find the most appropriate mutation level. Since the biological sequences have sequencing errors, the perturbance method also enables us to test the robustness of the tree obtained even though the sequences had experimental errors.

### Relative complexity measure distance method

RCM computes organisms’ relatedness based on the relational complexity of the sequences. Overall work flow as explained in [18] is as follows. The two step method begins with an exhaustive library of the primary sequence generated by the Lempel and Ziv method [20]. A new library is created from the second sequence by using the exhaustive library of the primary sequence as a starting point. At the heart of RCM lies calculation of the LZ-complexity of a sequence, which is obtained by counting the number of steps needed to generate a copy of the sequence starting from a null state. Each step involves a process of copying a nucleotide or a series of nucleotides for a sequence and then adding the next nucleotide from the sequence being analyzed. The number of steps needed to obtain the exhaustive library is identified as the LZ-complexity value of the given sequence. The LZ-complexity of “TGATGCGACACA” is obtained as an example in Table 2. Since six steps were needed to generate the exhaustive library, the LZ-complexity for the analyzed sequence is ‘6’. 

**Table 2:** Explanation of LZ-complexity of the sequence “TGATGCGACACA”

**Step**	**Copy**	**Add**	**Generated Sequence (Z)**
1	-	T	T
2	-	G	T.G
3	-	A	T.G.A
4	TG	C	T.G.A.TGC
5	GA	C	T.G.A.TGC.GAC
6	ACA	-	T.G.A.TGC.GAC.ACA

In each step of the LZ production process, copying takes place as much as possible from history followed by a single nucleotide addition. For example, in the first three steps shown in Table 2 there is no copy action; only single nucleotide addition is performed. Fourth step starts with search term T. Different from the first three steps, nucleotide T at current position can be copied from the history because T can be found at the first position in the sequence. While search term can be copied from history, current search term - which is T - will be expanded with the nucleotide at the next position. After addition of the next letter, search term becomes TG. Since the updated search term, TG, is also found in the history at position [1,2], search term will be expanded with the next nucleotide and will be searched again in the history. Updated new search term is TGC, but it is failed at search in the history. Fourth step ends with copying the last successful search term from the history and single nucleotide addition follows.

Given two sequences S_1_ and S_2_, RCM is defined as the number of steps required generating S_1_ from S_2_ as opposed the generating S_1_ from a null state. This measure, which defines a level of closeness between two sequences, forms the bases of a number of distance metrics that have been shown to be fit to use in phylogenetic analysis. Let c(S) denotes the LZ-complexity of the sequence S. We used the RCM distance between two sequences S_1_ and S_2_ as

(1) dS1,S2=cS1S2−cS1+cS2S1−cS212cS1S2+cS2S1

Here, S_1_S_2_ represents the concatenation of the two sequences S_1_ and S_2_; therefore, c(S_2_S_1_) – c(S_2_) reflects the number of steps required generating S_1_ from S_2_. The calculations were done using the original algorithm and software as previously described [18] and there were no disagreements between the topologies rendered by different RCM distance methods as previously reported [18,19,21].

### Tree construction method

We constructed a matrix of distances between all OTUs for each data set based on the aforementioned RCM distance method. Resulting matrices were used to reconstruct phylogenies using neighbor-joining (NJ) method in NEIGHBOR routine of Phylogeny Inference Package (PHYLIP version 3.67) [40]. Consensus of all output trees of NEIGHBOR were computed by CONSENSE routine of PHYLIP using extended Majority Rule (MRe). TREEDIST routine of the PHYLIP package was used to compute topology (symmetric) distances [41] between the original tree and the consensus tree of 1000 bootstraps for each mutation level. Branch lengths were not considered as significant in this study. Only topology distances were calculated to present the stability or change in the phylogeny.

In the comparison of RCM with other methods of phylogeny construction, RCM-NEIGHBOR, DNADIST-NEIGHBOR, DNAML and DNAPARS routines of PHYLIP were used. F84, GTR and K2 DNA models were used to create distance matrices by DNADIST. Default program parameters were used for rest of the processes.

## Results

### Validation of the RCM approach

Simulated data were generated based on two DNA Models with balanced topology. By using Robinson and Foulds‘ method of Symmetric Distance [41], average distances of 2000 sequence families for each of four phylogeny construction method were calculated (Table 1). The results of tree comparisons have shown that the trees constructed by RCM method were the closest to the true topology demonstrating the accuracy of the RCM method for both DNA models. POY and DNAML were the second in F84 and K2P models respectively.

### Real data

**Figure 3:**
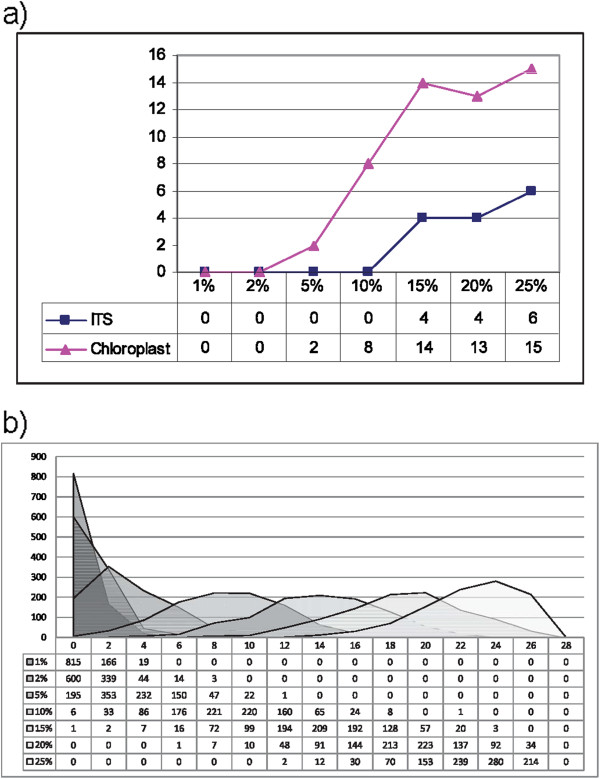
**Graph and Histogram based on of Tree distances at different mutation rates. a**) Tree distances between the trees of different mutation rates (1%, 2%, 5%, 10%, 15%, 20% and 25%) and the original tree. Trees resulted from nuclear ITS sequences keeps their topology up to 10% level mutation, while topology has changed after 2% in chloroplast sequences. **b**) Distributions (histograms) of the topology distances for only ITS sequences computed between the mutation trees and the original tree.

Phylogenies based on ITS and Chloroplast sequences were constructed using the RCM method with the application of the perturbation technique. The reliability and robustness of the RCM approach was tested by examining the impact upon topology integrity by progressively mutating portions of the molecular sequences. The RCM generated trees retained identical topology after mutating up to 10% of the nuclear ITS sequence and 2% of the chloroplast DNA sequence (Figure 3). The change in the topology of nuclear sequences was not as high as that of the change in chloroplast sequences.

**Figure 4:**
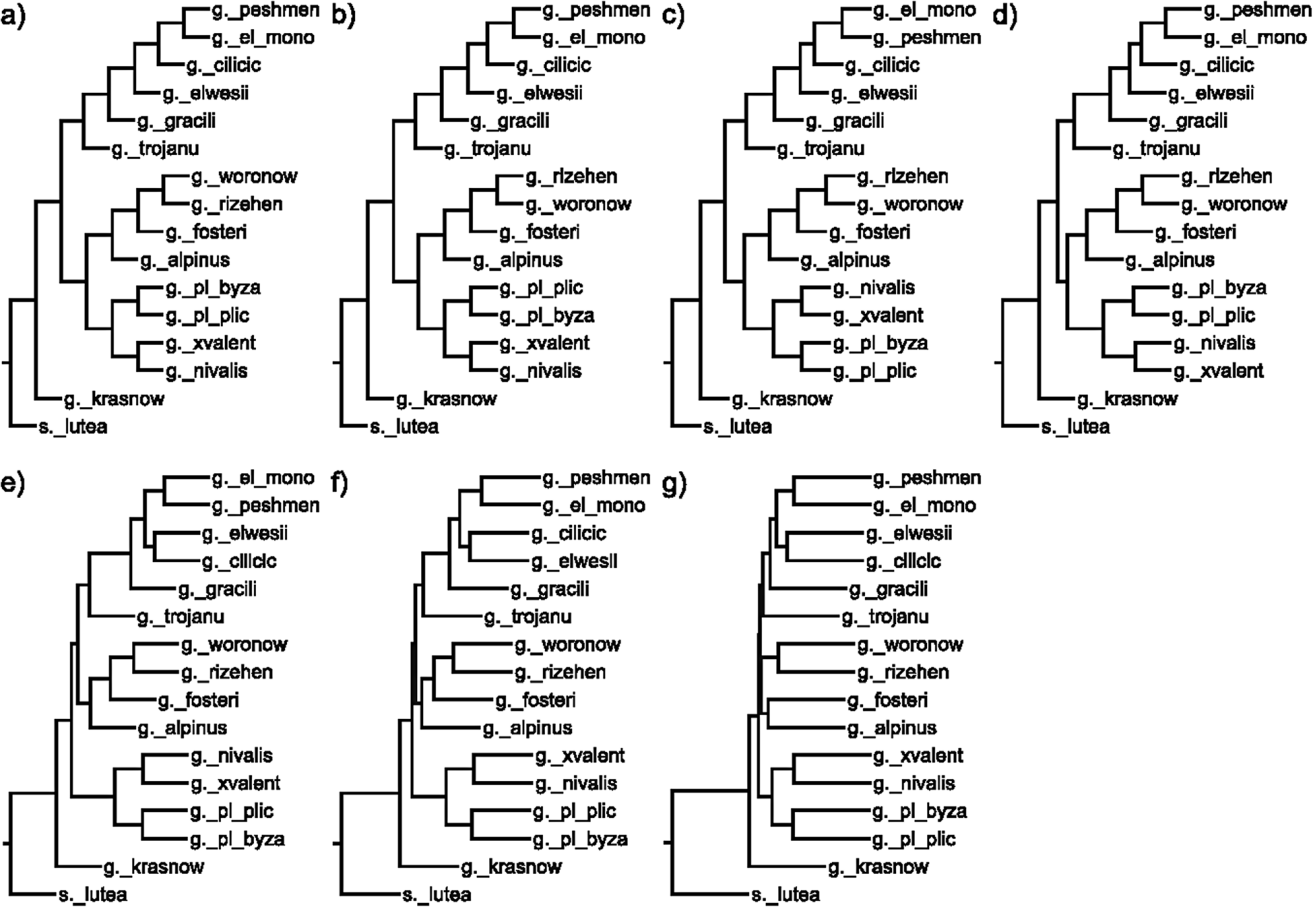
**ITS consensus trees belong to different mutation levels.** Consensus trees generated at mutation levels (**a**) 1%, (**b**) 2%, (**c**) 5%, (**d**) 10%, (**e**) 15%, (**f**) 20%, (**g**) 25% for ITS sequences. Numbers at branching points indicate the number of occurrences of a branch in trees obtained using individual resampled data sets.

**Figure 5:**
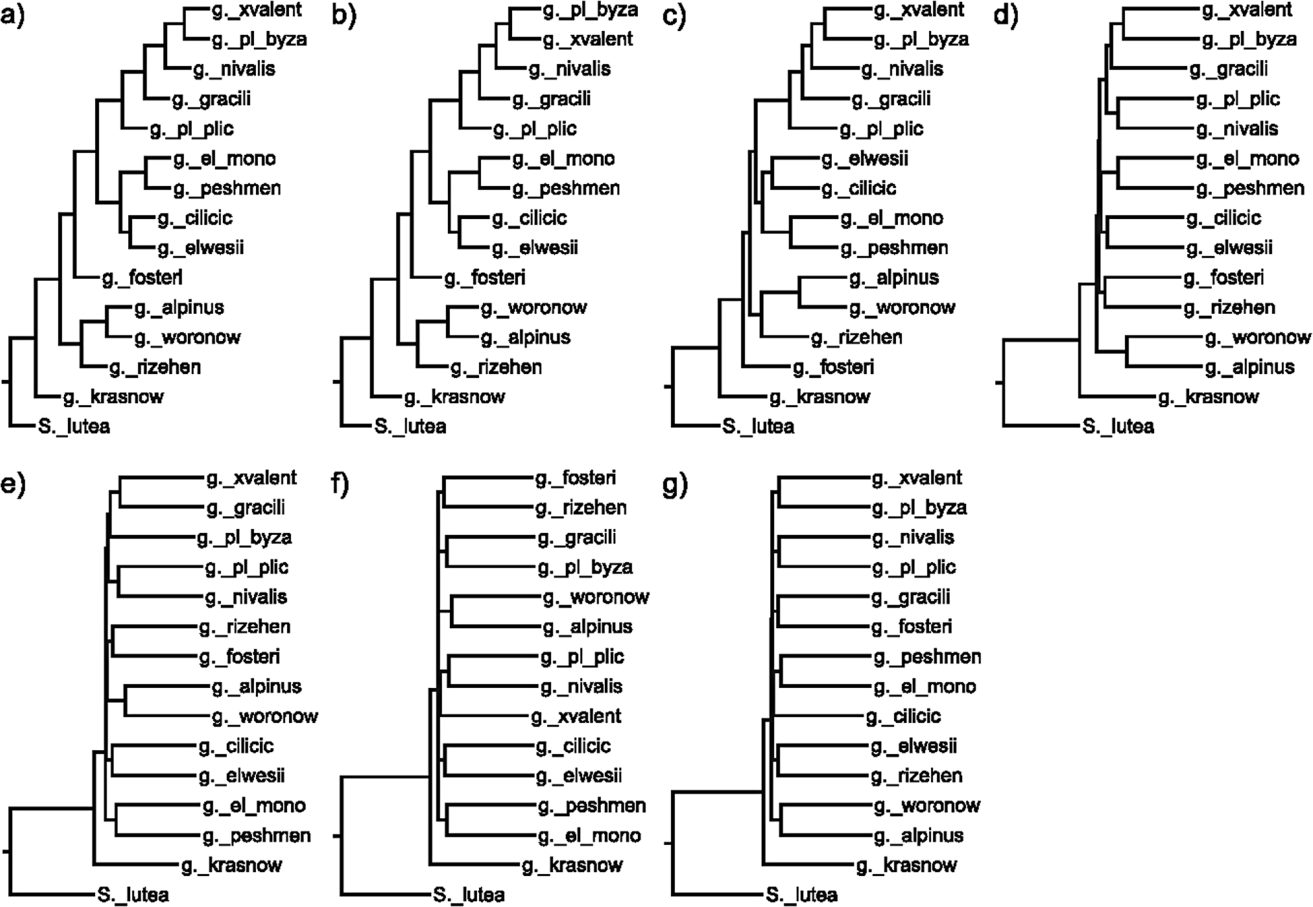
**Chloroplast consensus trees belong to different mutation levels.** Consensus trees generated at mutation levels (**a**) 1%, (**b**) 2%, (**c**) 5%, (**d**) 10%, (**e**) 15%, (**f**) 20%, (**g**) 25% for Chloroplast sequences. Numbers at branching points indicate the number of occurrences of a branch in trees obtained using individual resampled data sets.

In both nuclear (Figure 4) and chloroplast (Figure 5) based phylogenies, *G. krasnovii* was positioned as an outgroup in addition to *S. lutea*. All other species form three groups in ITS sequences. Even at higher rates of mutation, consensus trees of ITS sequences keep the same topology. Although branches differ inside and outside of these groups, species always show the same groupings. For the chloroplast sequences, the tree topology increasingly differs both from the original tree and from each other with the mutation rate. The change in topology becomes constant after 15% mutation rate through 25%.

The Maximum parsimony method gave the best resolutions to relationships among OTUs in trees constructed by using nuclear ITS sequences in [42]. Trees generated by Minimum evolution and Neighbor Joining methods beard unresolved relationships among taxa within the group and in the upper branches. However, both nuclear and chloroplast DNA sequences gave an exact resolution in RCM even at higher levels of mutations.

In the current study, *G. alpinus* always clustered together with *G. fosteri*, *G. woronowii* and *G. rizehensis*, all grouped under subseries *Viridifolii* (series *Latifolii*). This grouping is strongly supported by the molecular data of [47]. In their work, they had performed phylogenetic analysis of *Leucojum* and *Galanthus* species based on *mat*K gene and rRNA ITS sequences and reported that *G. alpinus* clustered on the same branch with *G. woronowii* and *G. fosteri*[47]. However, our predictions about the genetic relatedness of *G. alpinus* deviates from that of [29] which is based on morphological data of several taxonomists. In [29], *G. alpinus* is grouped with subseries *Glaucaefolii* together with *G. gracilis, G. cilicicus, G. peshmenii, G. angustifolius, G. elwesii* and *G. koenenianus*. In the current work, *G. elwesii, G. gracilis, G. cilicicus, G. peshmenii and G. elwesii var. monostyctus* formed a monophyllic group as well as in [47] and [42], according to their rRNA ITS sequence based phylogenetic trees. Series *Latifolii*, composed of subseries *Glaucaefolii* and subseries *Viridifolii*, has been represented as sister clades with a very low bootstrap confidence values (14, 16) with Minimum evolution and Neighbour Joining methods based on more discriminatory nuclear rRNA ITS region in [42] while Maximum Parsimony had resulted in sister clades of series *Galanthus* and series *Latifolii* subseries *Viridifolii* with bootstrap value of 75%. RCM produces a topology similar to the one that produced by Maximum Parsimony and it keeps producing the same topology even at higher mutation levels. The most morphologically distinct member of the genus *Galanthus* is *G. krasnowii*[48]. *G. krasnowii* formed a separate branch in the phylogenetic trees constructed in this study in accordance with [47] and [42].

The case of *G. trojanus* has been explained as “species of uncertain affinity” in [29]. In [42] it has been found to be in different positions according to the phylogenetic method applied. Maximum Parsimony approach places *G. trojanus* outside of all groups, whereas all the other methods locate it in the group series *Latifolii*[42]. In the current study, *G.trojanus* has been located closer to the series *Latifolii* subseries *Glaucaefolii* with higher confidence values even at higher levels of mutation.

### Comparison of phylogeny construction methods

When we compare phylogenies based on ITS sequences in Figure 4, neither ML, nor Parsimony analysis were able to resolve the relationships among series *Latifolii*. *G. cilicicus*, *G. elwesii* var. *elwesii* and (*G. elwesii* var. *monostictus* + *G. peshmenii*) were found in a monophyletic relationship in phylogenies of character-based methods. Relationships among all OTUs were clearly resolved in phylogenies of distance-based methods, F85, K2, GTR and RCM. None of the methods have claimed the taxonomy in Bishop et al. based on series and subseries. F85, K2, and GTR kept series *Galanthus* and series *Latifolii*-subseries *Glaucefolii* within the same branch, while RCM and character-based methods grouped series *Galanthus* and series *Latifolii*-subseries *Viridifolii*. However, Only RCM was able to locate *G. trojanus* into series *Latifolii*-subseries *Glaucefolii*.

In phylogenies based on Chloroplast sequences (Figure 5), all distance based methods except RCM have failed in calculation of branch lengths. As a result, neighbor-joining algorithm has assigned negative lengths to the branches. Although chloroplast sequences were unable to locate most of the taxa within the correct clusters, all of the methods have grouped series *Galanthus* and series *Latifolii*-subseries *Glaucefolii* closely as sister clades. Character-based methods have not resolved all the relationships among taxa as was the case in ITS data. For both data, RCM had generated phylogenies with fully resolved relationships and with reliable branch lengths. Only RCM was able to root the tree correctly from *S. lutea* while others located it as an inner branch within the output tree of phylogenies produced from Chloroplast (Figure 5).

**Figure 6:**
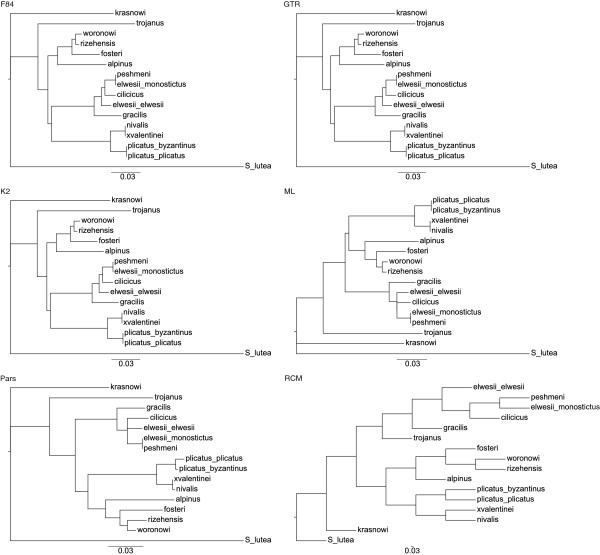
**ITS Phylogenies belong to different Phylogeny construction methods.***G. trojanus* was able to locate into series Latifolii-subseries Glaucefolii by only RCM. The method was also able to generate phylogenies with fully resolved relationships and with reliable branch lengths. RCM was the one which locate outgroup correctly. Measure for branch lengths were indicated below each tree.

Trees in Figure 6 have been constructed based on the topology distances between the trees generated by RCM (at different mutation rates) and the other methods. The results indicate that RCM created highly robust phylogenies. Even when an unrealistic value of 25% of the sequence has changed; the resulting phylogeny is still very close to the original (Figure 6).

## Discussion

RCM is an alignment-free molecular sequence distance measure that can be applied to phylogeny reconstruction, which is most useful when whole genome phylogenies or phylogenies based on more than one gene or protein are considered. As RCM builds a vocabulary over the given molecular sequence, its accuracy is jeopardized for short sequences. Moreover, existing approaches for alternative phylogeny construction methods where statistical significance is assigned to tree branches are not applicable to RCM. Our results on the simulated data sets indicate that even for relatively short sequences RCM is able to provide accurate trees. On the real data sets, RCM builds robust trees, successfully resolves the relationships among taxa with accurate branch lengths, and is free of potential ambiguities caused by a preceding MSA step.

**Figure 7:**
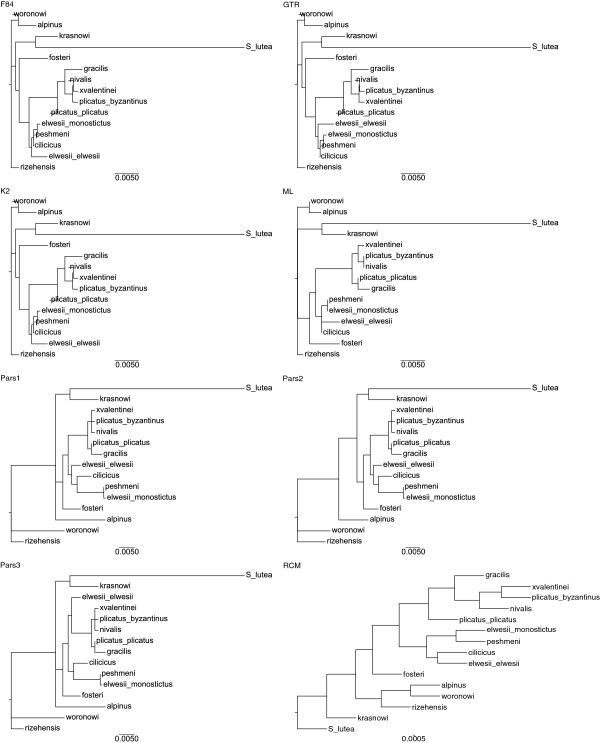
**Chloroplast Phylogenies belong to different Phylogeny construction methods.** Neither ML, nor Parsimony analysis were able to resolve the relationships among series Latifolii. *G. cilicicus*, *G. elwesii* var. *elwesii*. All distance based methods except RCM have failed in calculation of branch lengths. Measure for branch lengths were indicated below each tree.

**Figure 8:**
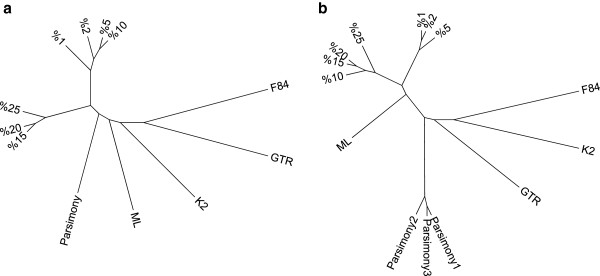
**Super trees based on ITS and chloroplast sequences.** Figure shows two super trees of (**a**) ITS sequences and (**b**) Chloroplast sequences based on compared methods. Each super tree was constructed from a distance matrix calculated by finding distances between the found topologies. The results indicate that RCM created highly robust phylogenies. Even when an unrealistic value of 25% of the sequence has changed; the resulting phylogeny is still very close to the original.

A perturbation technique was used to test the robustness of the RCM method based on biological mutations. Although the perturbation technique simulates biological mutations, it can also be considered as a resampling technique as the applied procedure maintains the base composition of the sequence (the A:T:G:C ratios within each sequence would be conserved). While degree of degeneration can be adjusted by performing mutations in percentages, un-realistic 10% to 25% mutations were performed to test robustness and degree of chance in topology. We know that in evolution the DNA mutation rate is really low among the same species. Although it is very hard to reconstruct the “true” phylogeny from such highly mutated DNA sequences, we have aimed to reach the true topology at the consensus tree. Results showed that the consensus topologies were highly consistent especially for the ITS data. The reason for that nuclear ITS sequences gave more consistent trees (Figure 7) compared to chloroplast sequences (Figure 8) is most possibly because chloroplast is of endosymbiont originate and inherited maternally.

Nuclear and chloroplastic DNA sequences were obtained from the same individual. Therefore, disagreement between ITS and chloroplast trees may be explained by hybridization. However, we don’t think that we can comment on hybridization of the species since there was not enough evidence to prove such an event.

Branch lengths calculated by RCM were more accurate compared to the other techniques. Presence of “0”s or negative values in branch lengths in phylogenies of all other techniques reduces the reliability of these techniques. Although a non-positive branch length does not necessarily affect the topology, it causes complete loss of branch length information for the OTU and decreases the reliability of the branch lengths of other OTUs.

Placement of *G. trojanus* in the systematics of *Galanthus* is not very well established [29]. Thus, proposed positioning of *G. trojanus* warrants future work on this new branching. We believe that further investigation on *Galanthus* subspecies with more individual samples in the region is required. This is especially important to clarify the genetic relationship of both *G*. *trojanus* being a geographically isolated, poorly known species and *G. gracilis*, representing a larger distribution range when compared to other *Galanthus* subspecies.

## Conclusions

Relative complexity measure has been introduced as a distance measure based on LZ complexity for phylogeny construction and has been tested by constructing different phylogenies. Particularly, to overcome MSA-based problems, RCM seems to be a reasonable way and a good alternative to MSA-based phylogenetic analysis. Although the phylogenies generated by RCM were found highly reasonable, the statistical significance of the obtained trees have never been tested in detail. Resampling techniques that have been used in phylogenetic analyses assess the statistical significance of the tree topology. However, most of these methods are based on data with equal-size samples or require a preceding alignment procedure and RCM does not rely on alignment. Therefore, in this study, we propose a perturbance technique to perform resampling operation. The proposed technique is mimicking the evolutionary process by simple mutations in order to test the robustness of the trees obtained by RCM. Initially, accuracy of the method was tested using different sets of benchmark sequence families. Mainly, robustness of the RCM approach was assessed on *Galanthus* molecular sequences which were from genetic material of two different cell organs telling different evolutionary stories. Results were compared to other phylogeny construction methods.

We believe our method will become a mainstream phylogeny construction method especially for the highly variable sequence families where the accuracy of the MSA heavily depends on the alignment parameters.

## Competing interests

The authors declare that they have no competing interests.

## Authors’ contributions

SY provided the botanical specimens, NT and NB participated in the extraction of molecular sequence data. HHO provided sequence distance measure. YB participated in the design of the study. YB and CM performed the statistical analysis. OUS and HHO conceived of the study, and participated in its design and coordination. All authors read and approved the final manuscript.
